# Parents' Need for Support From Family and Friends During Their Preterm Infants' Hospitalisation: A Cross‐National Qualitative Study

**DOI:** 10.1111/jan.16707

**Published:** 2024-12-30

**Authors:** Emma Kainiemi, Anna Axelin, Helle Haslund‐Thomsen, Rakel Björg Jonsdottir, Renée Flacking

**Affiliations:** ^1^ Department of Nursing Science University of Turku Turku Finland; ^2^ Paediatric Research Unit, Clinical Nursing Research Unit, Aalborg University Hospital, Clinical Institute Aalborg University Aalborg Denmark; ^3^ Faculty of Nursing and Midwifery University of Iceland Reykjavík Iceland; ^4^ School of Health and Welfare Dalarna University Falun Sweden

**Keywords:** family and friends, family‐centred care, neonatal intensive care, preterm infants, qualitative study, support

## Abstract

**Aim:**

To explore the need for support from family and friends among parents of preterm infants within neonatal intensive care.

**Design:**

A cross‐national qualitative study.

**Methods:**

In autumn 2019, 73 parents of preterm infants hospitalised in neonatal intensive care units in Denmark, Finland, Iceland and Sweden were interviewed. Data were analysed using reflexive thematic analysis.

**Results:**

The infant's hospitalisation created a complex context for parental support from family and friends. Social, emotional and practical support were crucial but often hindered by challenges related to the environment, timing, individual circumstances and relationships. Parents emphasised the need for family and friends to be supported in understanding the situation.

**Conclusion:**

Encouraging the presence and involvement of family and friends, implementing adaptive unit policies and sensitivity of staff are pivotal in fostering a supportive environment.

**Implications:**

By better understanding parents' need for support, neonatal units can further develop their practices and enhance the parental support. This can be achieved by enabling the presence and involvement of family and friends at the level the parents desire.

**Impact:**

This study addressed a knowledge gap regarding parents' support need from family and friends during their preterm infant's hospitalisation. This study's results will offer important knowledge to develop unit practices and comprehensive support systems for parents.

**Reporting Method:**

This study followed the relevant EQUATOR guidelines and COREQ checklist.

**Patient or Public Contribution:**

No patient or public contribution.

**Summary**

- What does this paper contribute to the wider global clinical community?
  ○ Facilitating family and friend involvement in neonatal care can enhance parental support and improve the well‐being of parents and their support networks.
  ○ Flexible policies on visiting hours and the roles of family and friends are vital to addressing parents' evolving support needs.
- What already is known:
  ○ Preterm birth and the infant's hospitalisation significantly impact parents' well‐being, causing stress, anxiety and depression that can hinder the parent–infant relationship, making support essential to mitigate these effects.
  ○ Family‐centred care emphasises the supportive role of family and friends in enhancing parents' well‐being and coping with the hospital environment.
  ○ Limited research exists on the role of family and friends in supporting parents during the infant's hospitalisation, with studies primarily focusing on healthcare staff or peer support, leaving gaps in understanding other support networks.
- What this paper adds:
  ○ Parents value the support of family and friends during their infant's hospitalisation, particularly for sharing experiences, maintaining a sense of identity beyond being parents of a preterm infant and alleviating practical burdens.
  ○ Parents' support needs change throughout hospitalisation and are influenced by the hospital environment, including unit culture and visiting policies, which shape the involvement of family and friends.
  ○ Parents highlight the need to provide family and friends with guidance and information to help them understand the challenges of preterm birth and offer meaningful support.
- Implications for practice/policy:
  ○ Neonatal intensive care units should establish clear and consistent guidelines aligned with family‐centred care principles to actively involve family and friends in supporting parents, enhancing their well‐being and fostering an easier transition to home.
  ○ Staff should implement flexible and responsive strategies to address parents' changing support needs and manage the involvement of family and friends according to parents' preferences throughout the hospitalisation.
  ○ Continuous reflection and adaptation of unit practices are necessary to ensure comprehensive and personalised support for parents and their support networks during hospitalisation and after discharge.

## 1 Introduction

The hospitalisation of a preterm infant in a neonatal intensive care unit (NICU) typically exposes parents to significant levels of stress, anxiety and depression (de Paula Eduardo et al. 2019; McKeown et al. 2023). NICUs around the world have adopted family‐centred care (FCC) practices, emphasising the crucial role of support in parental well‐being (Axelin et al. 2022). However, the staff have been observed to encounter difficulties in meeting the parents' support needs, and because the parents are encouraged to spend more time in the NICU, this issue may become increasingly problematic (Kainiemi et al. 2021). Despite the recognised importance of family and friends during a preterm infant's hospitalisation, studies indicate that they remain an underutilised source of support for parents, and the staff often do not consider facilitating such support part of their duties (Flacking et al. 2022). By gaining a deeper understanding of parents' support needs from their family and friends, NICUs can develop more comprehensive strategies to support parents during their infant's hospitalisation and enhance the inclusion of family and friends on the level desired by the parents.

## 2 Background

The preterm birth of an infant and the subsequent hospitalisation in the NICU often cause parents significant stress, negative emotions and anxiety (McKeown et al. 2023). Additionally, parents are at an increased risk of depression (Axelin et al. 2022), which can negatively impact their well‐being and the development of the parent–infant relationship (de Paula Eduardo et al. 2019). FCC is a care approach that aims to involve parents as active partners in their infant's care during NICU hospitalisation, potentially improving their well‐being and reducing the risk of depression (Ding et al. 2019). Although FCC encompasses diverse definitions and elements, the importance of support is emphasised (Kuo et al. 2012). Support is crucial for enhancing parental well‐being (Axelin et al. 2022), fostering strong parent–infant relationships (Amankwaa, Pickler, and Boonmee 2007) and helping parents feel like parents in a hospital environment (Adama et al. 2022).

Despite the recognised importance of support, previous research has shown that parents often feel inadequately supported by staff (Kainiemi et al. 2021) due to factors such as ineffective communication (Adama et al. 2022), lack of continuity in staffing and varying attitudes among staff (Wigert, Dellenmark, and Bry 2013). In addition, parents' support needs have been noted to evolve as NICUs are increasingly being architecturally renovated from shared patient rooms to single‐family rooms (SFRs) to support the implementation of FCC practices (Jones et al. 2016; Kainiemi et al. 2021). With parents being more present in SFR environments in the privacy of their own rooms (Jones et al. 2016), their need for support may be heightened (Kainiemi et al. 2021).

Given the potential limitations in staff support for parents and the potentially heightened need for parental support during their infant's NICU stay, it is crucial for parents to receive support from their extended network of close persons, such as family members and friends (Flacking et al. 2022; Kavanaugh et al. 2014; Smith et al. 2012). Both staff and parents have emphasised that parents want to share their experiences with and rely on for support from family members and close friends who are integral parts of parents' lives (Flacking et al. 2022; Hagen et al. 2019). According to the principles of FCC, individuals have the right to define their own support networks (Institute for Patient‐ and Family‐Centered Care 2024) and caregiving of the infant within a supportive family and community network is emphasised (Kuo et al. 2012). Therefore, it is essential to recognise not only family but also close friends as integral parts of the parent's support network during the infant's hospitalisation (Brødsgaard et al. 2017; Kainiemi et al. 2022).

Although the importance of family and friends for parents during their preterm infant's NICU stay is acknowledged and research has reported that family also expresses a desire to be engaged and supports the parents during the infant's hospitalisation (Adama, Bayes, and Sundin 2018; Brødsgaard et al. 2017; Frisman et al. 2012; Hall 2004; Lindberg and Ohrling 2008; Ravindran and Rempel 2011), many NICUs limit their presence. For instance, it is a common practice in NICUs to restrict the number of visitors and the time they are allowed to spend in the NICU, and family and friends are often permitted to visit only when accompanied by parents (Flacking, Breili, and Eriksson 2019). Although staff often recognise the important role of family and friends in supporting parents, facilitating this support may not always be seen as part of their responsibilities (Flacking et al. 2022), and their involvement in fostering these relationships can sometimes be limited (Smith et al. 2012). Some studies also suggest that some staff may not actively encourage parents to seek support from family and friends during their infants' hospitalisation (Shimizu and Mori 2018).

Research on the role of family and friends in supporting parents with a preterm infant hospitalised in the NICU is limited, particularly from the perspective of the parents themselves. Existing studies on support have largely focused on interactions between healthcare staff and parents (Jones et al. 2016; Shimizu and Mori 2018) and on peer support among parents (Rossman, Greene, and Meier 2015), with considerably less attention paid to the role of family and friends. When family and friends' involvement has been examined, the focus has often been on grandparents supporting the parents (Amankwaa, Pickler, and Boonmee 2007; Frisman et al. 2012; Hall 2004; Ravindran and Rempel 2011), whereas the role of other family members and friends has been minimally explored (Flacking et al. 2022; Smith et al. 2012). By gaining a deeper understanding of parents' support needs from their family and friends, comprehensive support for parents during their infant's hospitalisation can be enhanced. Therefore, the aim of this study was to describe the need for support from family and friends among parents of preterm infants during their infant's hospitalisation.

## 3 Methods

### 3.1 Design

This study was grounded in the principles of family systems theory and social support theory. Family systems theory emphasises the interconnectedness of family members and the impact of one member's experience on the entire family unit (Bowen 1985), and social support theory highlights the critical role of support in coping with stress (House 1987).

A qualitative descriptive methodology was chosen for this study because it captures the phenomenon in its natural setting, providing rich, authentic descriptions grounded in participants' own words and experiences (Bradshaw, Atkinson, and Doody 2017). This approach allows for a deeper understanding of parents' support needs and the roles of family and friends.

### 3.2 Participants and Setting

The study was conducted across four Nordic countries, where parents of preterm infants were interviewed. A total of seven NICUs in Denmark (*n* = 2), Finland (*n* = 2), Iceland (*n* = 1) and Sweden (*n* = 2) were selected to ensure a broad range of perspectives among parents. A convenience sample of potential participants was identified through collaboration with nurses working in the participating NICUs. Researchers directly collaborated with nurses to identify parents who could be approached, informed about the study and asked for their willingness to participate. Eligibility criteria included parents who were at least 18 years old and proficient in the country's native language. Parents whose infants were critically ill were excluded. Eligible parents were provided with oral and written information about the study, emphasising the voluntary nature of participation and their right to withdraw at any time. Those who agreed to participate were inquired about their preferred time and location for the interview. Additionally, the possibility for a phone interview was offered.

The participating NICUs provided care on levels III–IV. At the time of the data collection, six of the participating NICUs were a combination of SFRs and shared patient rooms, and one exclusively offered SFRs. Parents were encouraged to be present in all units, with reclining chairs and/or beds provided to facilitate their presence. In two NICUs with SFRs, at least one parent was encouraged to be continuously present 24/7 with the infant. In another NICU, parents were allowed to be with their infant during the day without the opportunity to stay overnight. It is noteworthy that the social welfare systems in the participating countries support parents' continuous presence in the NICU by offering paid parental leave for 9 months or longer.

All but one of the NICUs had a policy regarding visitations by family and friends. Most units had no visitor restrictions in SFRs, whereas in shared patient rooms, the number of visitors at a time was typically limited to two to ensure privacy. This limitation could be negotiated, depending on individual circumstances, such as other families' presence, the infant's health condition and the anticipated length of the infant's hospitalisation. In all units, parents had the freedom to invite whomever they wished to visit, but in some units, this was conditional on the parents being present.

Although most NICUs had written guidelines for the presence of family and friends, none specified their role and involvement in the care process. In the units where parents were encouraged to be present at all times, family and friends were recognised as crucial for temporarily assuming the infant's caregiving responsibilities. Conversely, in another unit, family and friends were restricted from activities other than holding the infant to prioritise bonding with the parents. In most units, family and friends' involvement was not restricted, permitting them to engage in activities such as skin‐to‐skin contact, feeding and diaper changes with the parents' consent.

### 3.3 Data Collection

The semi‐structured interviews were conducted in autumn 2019 during a 5‐month period. The interviews followed the interview guide (Supporting Information S1), which researchers from each respective country (HH‐T, AA, RBJ, RF) developed in English. The interview guide included questions regarding the parents' experiences of their support needs from their family and friends and how they preferred to involve them during the infant's hospitalisation. The semi‐structured interview guide was organised into main themes and follow‐up questions: main themes framed key research areas, whereas participants were encouraged to freely share their perceptions and experiences for an in‐depth exploration (Kallio et al. 2016).

After the interview guide was agreed on, it was translated into the participating countries' national languages (Danish, Finnish, Icelandic, Swedish) and thorough discussions were held to confirm that the meaning of the interview questions remained unchanged after translation. The first interview in each country was considered a pilot, and upon review of these pilot interviews, it was determined that no adjustments to the interview guide were required.

All the interviews were arranged face to face in the quiet and private surroundings in an NICU environment during the infants' hospitalisation. Some parents participated in interviews together with their partners, whereas others participated individually. Interviews were recorded with the participants' consent. The researchers maintained field notes to document their thoughts and observations. The duration of the interviews ranged between 14 and 80 min, with a mean of 32 min. Subsequently, the interviews were transcribed verbatim.

### 3.4 Ethical Considerations

The study was conducted in accordance with the Declaration of Helsinki and the guidelines for responsible research conduct by the Finnish National Board on Research Integrity (Finnish National Board on Research Integrity TENK 2023). Ethics approval was obtained from ethical committees in Denmark (Danish Data Protection Agency, 2008‐58‐0028), Finland (Ethics Committee at the University of Turku, 35/2019), Iceland (The National Bioethics Committee, VSN‐19‐108) and Sweden (the Swedish Ethical Review Authority, 2019‐01405). Additionally, approval was obtained from each participating hospital in accordance with local policies.

Prior to participation, all participants provided informed written consent and were assured of the voluntary nature of their involvement, as well as their anonymity. To ensure confidentiality, each participant was assigned a unique code to protect their identity. These codes were used throughout data collection, analysis and reporting, ensuring that no personal information was disclosed. Additionally, all identifying details were removed from the transcripts (Kaiser 2009). Data, including audio files, transcripts and field notes, were never transferred between countries but securely stored in password‐protected files in each country, following the General Data Protection Regulation (GDPR) guidelines for secure data management.

### 3.5 Data Analyses

The qualitative data were analysed using reflexive thematic analysis (Braun and Clarke 2021). We chose reflexive thematic analysis for its strength in capturing diverse personal experiences and social processes, offering flexibility in examining how individuals interpret their environment and support needs (Braun and Clarke 2024). The analyses followed six distinct phases: (1) familiarising oneself with the data, (2) generating codes, (3) constructing themes, (4) reviewing potential themes, (5) defining and naming themes and (6) producing the report (Braun and Clarke 2024). The analyses started after all interviews had been conducted in each country to ensure that preliminary analyses did not guide the interviews.

Initially, each researcher extensively reviewed the interview transcripts specific to their respective countries and familiarised themselves with the data. Researcher EK began the process by inductively generating initial codes from the Finnish data, identifying patterns of shared meaning across the dataset. A total of 45 initial codes were generated, capturing various aspects of parents' support needs during their infant's NICU stay. Subsequently, researchers HH‐T, RBJ and RF deductively coded the data from Denmark, Iceland, and Sweden while also suggesting additional inductive codes. Throughout the coding process, the research group engaged in active discussions and collectively reviewed the codes multiple times. The codes were grouped into categories, leading to the identification of three key themes and nine subthemes (Table 2) that reflected central aspects of participants' experiences. In this process, codes and categories were added and revised based on each researcher's individual coding. The themes were then reviewed and refined through ongoing team discussions. Each researcher revisited both the codes and the entire dataset from each country in joint discussions to ensure that the themes were coherent and accurately represented the participants' experiences.

Finally, the research group generated an initial storyline to describe the content of the themes. Variations observed in the data across countries were thoughtfully integrated into this storyline. To enhance transparency, detailed direct quotes from the interviews were included to enrich and complement the storyline (Lingard 2019). To enhance the quality of reporting, we utilised the consolidated criteria for reporting qualitative research (COREQ) checklist (Tong, Sainsbury, and Craig 2007).

### 3.6 Trustworthiness

The research team remained aware of how their interpretations of the phenomenon could influence the interviews, data analysis and reporting. Reflexive thematic analysis inherently emphasises that subjectivity influences data interpretation and is seen as a valuable resource for producing knowledge (Braun and Clarke 2021). The research team, comprising both experienced and less experienced researchers from different countries with diverse backgrounds in healthcare and neonatal care, enriched the analysis with varied perspectives.

Reflexive discussions during the process helped to ensure that their backgrounds and assumptions were critically examined, fostering a consistent understanding of the phenomenon. An audit trail of coding decisions and theme development enhanced confirmability by allowing others to trace the research process and confirm that the findings genuinely reflected the participants' experiences (Bradshaw, Atkinson, and Doody 2017). Additionally, the use of a rigorously developed and translated semi‐structured interview guide (Kallio et al. 2016) helped to ensure consistency across all interviews. Transferability was supported by providing detailed descriptions of the participants' experiences, allowing readers to determine the applicability of the findings to other settings (Bradshaw, Atkinson, and Doody 2017).

## 4 Findings

### 4.1 Demographic Information of Participants

A total of 73 parents participated in the study. The participants included 41 mothers and 32 fathers or partners. These parents comprised 43 family units with 37 singletons and six sets of twins or triplets. The participants' age ranged from 21 to 43 years, with an average age of 30 years. Most participants had a primary or secondary level of education. Approximately one‐fourth of the participating parents had older children at home. The parents on average lived approximately 63 min away from the hospital. At the time of the interviews, most participating parents slept in the hospital either with or without their infant in the same room. The infants were born between gestational weeks 23 and 36, with an average of 31 weeks. All infants had been hospitalised for at least 6 days before the interviews, with a median of 18 days. Table 1 provides the number of participants and their characteristics by country.

**TABLE 1:** Participants and their characteristics by country.

Participant characteristics	Denmark	Finland	Iceland	Sweden
Participants				
Mothers	8	13	10	10
Fathers/partners	8	7	9	8
Family units	8	14	10	11
Infants	10	16	11	13
Characteristics of the participant's infants				
Gestational age (weeks), *mean (range)*	33.0 (30.7–36.6)	29.4 (23.9–37.0)	31.6 (27.0–36.3)	33.5 (26.0–36.2)
Parity				
Singleton	6	13	9	9
Twins or triplets	4	3	2	4
Characteristics of the participating parents				
Age (years), *mean (range)*	28 (24–33)	31 (21–43)	29 (23–39)	30 (23–41)
Education				
Second level or lower	14	12	7	11
University degree	2	8	9	7
Older children at home	2	11	3	5
Time from home to hospital (min), *mean (range)*	28 (15–50)	37 (4–147)	30 (5–360)	139 (45–400)
Slept most of the time in hospital	14	4	10	18

### 4.2 Main Findings

#### 4.2.1 Complex Context for Receiving Support

Our findings showed that the NICU hospitalisation of a preterm infant constituted a complex context for parents to receive support from their family and friends. Parents expressed a strong need for and great appreciation of social, emotional and practical support from family and friends, which helped them cope during the infant's hospitalisation. Parents' support needs varied due to the challenging circumstances, making it difficult at times for their family and friends to know how and when to provide support. Recognising the complexity of the circumstances for their supporters, parents also considered it essential that their family and friends received support in understanding the situation. The findings are presented in three themes (Table 2).

**TABLE 2:** Themes, subthemes and codes in the overarching theme ‘complex context for receiving support’.

Themes	Subthemes	Codes
The needs for different types of support	Social support	Company
		Normalisation
	Emotional support	Sharing experiences
		Reassurance
		Empathy
		Empowerment
	Practical support	Assisting with daily household chores
		Financial aid
		Caring for older children at home
Varying support needs in challenging circumstances	Environment	Culture
		Schedules
		Architecture
	Timing	Admission
		Discharge
		Prolonged hospitalisation
	Parents' individual life circumstances	Health status
		Diverse life situations
	Relationship with family and friends	Dynamics between individuals
		Redefining family and friends
Supporting family and friends understand the situation	Managing interactions and knowledge transfer with family and friends	Understanding the situation by family and friends
		Facilitating the level of interaction
		Sharing information
		Various methods to ease interaction
	Providing emotional support for family and friends	Protecting feelings of family and friends
		Supporting family and friends emotionally

#### 4.2.2 The Needs for Different Types of Support

The theme ‘The needs for different types of support’ encompasses three subthemes, each addressing a distinct aspect of support: social, emotional and practical. Together, these subthemes highlight the various forms of support that were considered essential by the participants.

Parents expressed that the *social support* they received from their family and friends in the form of having company and engaging in conversations provided a brief break from the demanding hospital environment and helped them momentarily turn their attention to something else. Social interaction gave the parents a chance to experience moments of ‘normal life’, allowing them to feel like themselves in addition to being parents of a preterm infant. The parents perceived social support as revitalising, and it gave them the energy to cope with everyday life in the NICU.

Things here mostly revolve in such a small, self‐contained circle and in a bit of a bubble. When someone comes to visit, they usually talk about other things as well, which is quite refreshing and provides a welcome change. Because the days here follow a fairly consistent rhythm and level, it becomes like Groundhog Day, so you don't really know what day it is. Therefore, if someone comes to visit, that's what then marks the day. FIN

Parents found *emotional support* from family and friends incredibly important. Many shared that discussing their experiences with family and friends brought them comfort because they felt more at ease sharing personal matters with them than with the NICU staff. Parents appreciated when their family and friends demonstrated empathy by listening to their fears and anxieties and offering understanding and comforting words. This reassurance helped reduce parents' concerns about the situation, such as the infant's health and future development. Emotional support from family and friends was empowering for the parents, giving them the strength to face and overcome the challenges of the NICU more effectively.

It's the mental bit, to share your thoughts and get good answers. To balance your feelings. A friend knows who I am as a person and my weaknesses and can balance those things so that I don't get into low. SWE

Although parents mostly appreciated and relied on emotional support from their family and friends, there were times when their needs were not met, leaving them feeling disappointed and excluded. Occasionally, family and friends unintentionally had said hurtful things, which made the parents feel worse. Parents mentioned that sometimes their family and friends shared stories about premature infants and compared their situation to others. This made parents uncomfortable because it often seemed to undermine their unique challenges. The parents noted that it was important that their family and friends maintained a positive image of the infant and the circumstances, for excessive lamenting and underemphasis of the seriousness of the situation made the parents feel hurt and deepened their distress.

I told (to family member) that I am nervous about the infant's upcoming surgery day, and the only comment I got was, like, why to worry about such things … It felt dismissive and really hurt my feelings. FIN

*Practical support* from family and friends, such as helping with daily chores, including cleaning, laundry, meal preparation and taking care of pets, was very valuable for parents. Some parents mentioned that they received financial support to help them cope while in the hospital. Parents with older children at home felt grateful when family and friends offered to help care for their older children so that they could stay together at the NICU. It eased the parents' feelings of guilt when they knew that their older children were in the company of someone with whom they felt comfortable and enjoyed spending time. Some parents did not receive this much needed practical support, which added to their stress because they had to manage and sort things out on their own during an emotionally challenging time.

### 4.3 Varying Support Needs in Challenging Circumstances

The theme ‘Varying support needs in challenging circumstances’ comprises four subthemes: the environment, timing, parents' individual life circumstances and relationships with specific family members or friends. These subthemes illustrate the different aspects influencing the support needs of parents in challenging circumstances of their infant's NICU hospitalisation.

Parents expressed that numerous aspects regarding the *environment* complicated the situation and caused variation in their support needs and their family and friends' ability to provide support. They stated that the staff's attitude significantly influenced the extent to which the involvement and presence of family and friends was allowed and encouraged. When visitations were restricted to specific times, it limited the presence of family and friends, which made it harder for parents to receive the support they needed.

I am quite OK with the controlled visitations of family and friends in the unit, but maybe there is too many limitations. It would be nice to occasionally have visitors in the morning and not only in the afternoon. I also find it quite limiting that only two visitors are allowed at the same time. Many family members are left out, you don't want to choose between your family members. ICE

Some parents also emphasised that these restrictions offered a sense of comfort and familiarity in an otherwise uncertain situation. The restrictions the unit established also gave the parents relief because they did not have to enforce restrictions on their family and friends' presence or participation. Instead, they could refer to the restrictions the unit had set.

The unit's architectural layout influenced parents' need for support and their family and friend's capabilities to provide support while visiting the parents and the infant. Parents in SFRs appreciated having their family and friends around in the privacy of their own rooms. Parents whose infants were in shared patient rooms felt that this layout presented challenges, particularly when multiple visitors were present simultaneously; it was not always feasible to meet guests in other areas of the unit, and the designated parents' lounge lacked privacy or was limited to parents.

Parents also expressed that *timing* played a crucial role in their need for support, and their needs for the type and amount of support evolved during the infant's hospitalisation. Many expressed that at the beginning of the infant's hospitalisation, they desired to process the situation privately and did not have the energy to reach out to their family and friends.

I stopped people from coming in the beginning, I felt my head was spinning and it was too much to take in. I'm very much the ‘hostess’ normally and I didn't want to end up serving coffee. SWE

As the infant's health stabilised and the need for medical equipment decreased and after parents had sufficient time to process their emotions privately, they felt more prepared to share their emotions with family and friends and expressed a growing need for emotional support. Conversely, practical support was perceived as highly important and valuable throughout the hospitalisation, particularly regarding the care of older children. Moreover, if the infant's hospitalisation was extended, parents often increasingly relied on practical support as household chores piled up and needed attention. Some parents believed that their need for support would be greater after discharge as the NICU provided a structured environment with staff available to support them.

When we go home with her, it's a completely different thing with the need for support because, in a way, we are really well taken care of and don't really lack anything here. FIN

*Parents' individual life circumstances* also contributed to variations in their support needs. Parents expressed that aspects such as living far from the hospital, being a single parent and having older children at home impacted their ability to be present in the NICU and especially affected their need for practical support from their family and friends. In units requiring constant parental presence, parents valued the companionship of family and friends, which allowed them to take breaks and provided valuable assistance and companionship.

She (grandmother) is here so that we can leave for a while. Because we have a three‐year old at home, we take turn to be here and then we are mostly alone. Just to have the company means the world. Just to talk with someone about ordinary things. Just being here with you, maybe getting a sandwich for you. And to take over for a while. But just being there, that's the most valuable. SWE

Parents also recognised that their *relationships with particular family members or friends* shaped their support needs, resulting in specific expectations for how these individuals should provide support. Unmet expectations could lead to conflicting feelings and distancing in the relationship. The challenging period of the infant's hospitalisation sometimes changed the dynamics between parents and their family and friends; some relationships grew closer, whereas others became more distanced. The parents' narratives also made evident that they often felt more connected with and appreciative of those who provided support or had gone through a similar experience, which strengthened these relationships through shared understanding.

I have a friend who also has had a preterm child, and she has shown great interest and really understood the whole situation. I have used her to ask a lot of questions and it was so useful that she knew what I was talking about. DEN

### 4.4 Supporting Family and Friends Understand the Situation

The theme ‘Supporting family and friends understand the situation’ consists of two subthemes: managing interactions and knowledge transfer with family and friends, and providing emotional support to them. Together, these subthemes highlight the need that parents keep their family and friends informed about the situation in the NICU while also providing them with emotional support.

Parents strongly emphasised that to receive support, they also needed to help their family and friends understand the situation. Many parents *facilitated interactions and knowledge transfer* with their family and friends, recognising the importance of information and involvement. Most parents expressed a desire to maintain control over the information shared about their infant's condition and preferred to be the ones directly communicating with family and friends. To ease the burden of updating multiple family and friends, parents described how they sometimes sought assistance. For example, they asked grandparents to communicate with extended family members or used group discussions on mobile apps. This reduced the pressure of individual updates for family and friends because parents could inform multiple people simultaneously.

We used this group messaging app in our mobiles, where my friends' shared updates with each other if they had heard new information from me over the phone. This way, I didn't have to worry about informing each person individually. ICE

It was prevalent in many parents' narratives that they assumed the role of supporter partly due to their perceived obligation to involve their family and friends. Several parents described how they balanced between receiving and giving and felt a reciprocal need to interact with their family and friends. Moreover, many parents expressed a pervasive feeling of owing something to their family and friends for the provided support. This sometimes led to pressure to always be available to their family and friends, which in turn caused stress and feelings of being overwhelmed among parents.

You kind of feel that you owe them to be allowed to be a part of it all, because it is so kind and well meant – but it has also stressed me, and I was quite occupied already with the tasks here in caring for our daughter. DEN

Parents acknowledged that the situation was entirely new and unfamiliar to their family and friends, who often had varying levels of understanding about prematurity and its associated challenges, as well as the emotional burden parents face. Parents perceived that this sometimes led to a gap in recognising their need for support and knowing how to provide it effectively.

It has sometimes been a bit difficult to explain to friends, what this environment really is, what actually happens here. Like, for example, one of my friends genuinely asked that what do I do there all day? When they have seen the situation here, this environment, and … and perhaps that certain kind of, could you say, our tiredness in here. That's when it perhaps becomes more clear for them what life is like here. FIN

Parents recognised the difficulty and stress their family and friends faced and sought to *provide emotional support* to them during challenging times. They felt responsible for safeguarding them from distressing news, protecting their feelings and easing excessive worry. Parents whose family and friends spent a lot of time in the NICU emphasised these individuals' need for support, for they were deeply involved in NICU life and experienced its challenges first hand.

Some parents emphasised that their family and friends would understand the situation and parents' support needs better if staff would provide them with relevant information and support.

I think it would be great if the staff gathered the closest ones to the family and describe that the hospital stay can be long, and the family needs a lot of support during that time. This and this can be supported. Then I think it would help. Because it's really hard to ask for help. And many don't understand how difficult it is to be in this situation and that a little help make a huge difference. SWE

## 5 Discussion

In this qualitative, cross‐national study, parents of preterm infants hospitalised in NICUs across four Nordic countries were interviewed to describe their need for support from their family and friends. According to our findings, parents needed and valued various types of support from their family and friends. Their support needs varied due to the challenging circumstances, which sometimes, according to the parents, made it difficult for their family and friends to understand when and how to provide support. Recognising the circumstances' complexity for their family and friends, parents also believed it was essential that their supporters were supported in understanding the situation.

In our study, parents emphasised the importance of sharing their experiences, fears and anxieties with family and friends. Family and friends had a pivotal role in normalising the situation, which helped the parents retain their sense of identity beyond being parents of a preterm infant. This aligns with previous research, which underscores family and friends' importance in this process from the staff's perspective (Flacking et al. 2022). Parents also highly valued practical support, which alleviated stress from household chores and caring for older children, enabling more presence in the NICU. These findings align with prior studies underscoring the need for engagement of family and friends to mitigate negative experiences and facilitate the transition to parenthood (Amankwaa, Pickler, and Boonmee 2007; Frisman et al. 2012; Hall 2004; Lindberg and Ohrling 2008; Ravindran and Rempel 2011; Smith et al. 2012). However, some parents reported that their family and friends did not adequately address their support needs, thereby making the parents experience emotional stress and disappointment, aligning with the results of previous research (Kavanaugh et al. 2014; Smith et al. 2012).

In our study, parents highlighted the NICU environment's significant impact on their support needs and their family and friends' ability to provide support. Specifically, they emphasised the prevailing culture's influential role in the unit, which shaped how staff perceived the roles of family and friends and determined the extent of their involvement. Unit culture encompasses the social contexts that influence staff behaviour and establish accepted norms in the unit (Leep‐Lazar and Stimpfel 2024). Parents in our study noted that this culture extended to visiting policies, among which significant variations have been shown even within the same countries (Flacking, Breili, and Eriksson 2019). It is crucial to assess whether these restrictions in visiting and involvement of family and friends in the caretaking of the infant are truly necessary. Consistent unit policies are needed in NICUs regarding the integration of family and friends in the caretaking of the infant at levels determined by the parents (Flacking et al. 2022). Additionally, unit guidelines should emphasise the importance of staff remaining attentive to parents who lack a support network among family and friends (Hagen et al. 2019) and who may rely on the staff as their sole source of support in times of need.

Many parents emphasised that their need for support from family and friends changed throughout their infant's hospitalisation. They expressed that after the preterm birth and the infant's hospitalisation, they needed time to process the events privately and gather their thoughts and strength, primarily needing practical support, as noted in previous research (Lindberg and Ohrling 2008). Additionally, parents highlighted that being in the NICU 24/7 increased their need for social, emotional and practical support from family and friends to help them cope with the challenging circumstances. This aligns with research indicating increased support needs for parents in SFRs (Kainiemi et al. 2021). Parents also emphasised the likelihood of increased support needs from their family and friends after discharge. Staff should acknowledge family and friends' crucial role in families' lives during the hospitalisation and post‐discharge and aim to empower them to offer parents appropriate support (Adama, Bayes, and Sundin 2018). Enabling the involvement of family and friends in the NICU can ease the transition home for parents and facilitate the support they receive because family and friends have opportunities to bond with the infant (Flacking et al. 2022). Therefore, there is a need for ongoing reflection on NICUs on how to effectively support not only parents but also their family and friends during the infant's hospitalisation and after the transition to home.

Parents recognised that the unique circumstances of prematurity and infant's hospitalisation were often unfamiliar to their family and friends, who lacked a comprehensive understanding of the challenges parents face. This aligns with previous research indicating that premature birth and infant hospitalisation typically present unfamiliar territory for family and friends, resulting in potential gaps in understanding how to offer adequate support and uncertainty about their role in supporting parents (Frisman et al. 2012; Kavanaugh et al. 2014). Previous research has indicated that staff have observed that being physically present in the NICU and experiencing the daily challenges parents face can help family and friends develop a deeper understanding of those challenges, thereby enhancing their ability to provide meaningful support (Flacking et al. 2022). Additionally, staff should ensure that family and friends receive relevant information on prematurity and parental support needs (Frisman et al. 2012; Ravindran and Rempel 2011) through organised group sessions or guidance to appropriate sources of information.

### 5.1 Strengths and Limitations

Our study included a substantial number of parents with varying demographic backgrounds from four Nordic countries. Their preterm infants were hospitalised in NICUs with diverse architectures, FCC practices and varying policies regarding the involvement of family and friends. In addition, our study focused not only on mothers' experiences but also on those of the fathers and partners who have historically been underrepresented in research.

The inclusion of 73 participants from various countries aimed to capture a wide array of parents' experiences and perspectives. Considering the cultural differences and NICU practices among these countries, we sought to ensure that our analysis comprehensively reflected this diversity. In line with reflexive thematic analysis, our focus was not on reaching saturation but on generating a rich, nuanced understanding of the data (Braun and Clarke 2021). We included participants until we were confident that the country‐specific data accurately represented parental experiences within each country. This approach allowed for a broad examination of commonalities and differences across countries and contexts when the data were combined, thereby enhancing the transferability of our findings.

One limitation of our study is that we employed convenience sampling rather than purposive sampling, which might have influenced the representativeness of the sample (Bradshaw, Atkinson, and Doody 2017). Nonetheless, the diversity in participant's backgrounds in terms of age, education level, relationship status, and the characteristics of their infants enabled us to capture a wide range of experiences. The presence of both positive and negative experiences regarding support from family and friends indicates a wide range of perceptions among the participants. This diversity supports our findings' applicability and transferability to other settings.

It is important to note that in the Nordic countries participating in this study, the culture and architecture of NICUs, along with comprehensive family leave policies, enable parents to be continuously present in the NICU. These structural advantages may significantly impact the overall care process and parents' experiences, which should be considered when applying our findings to other countries with different NICU practices and family leave policies.

## 6 Conclusions

In conclusion, recognising the vital role of family and friends in supporting parents of preterm infants is essential for fostering a more inclusive and supportive NICU environment. Actively involving family and friends in the care process can enhance the well‐being of parents and their support networks and ensure continuous support after discharge. As parents' support needs evolve throughout the infant's hospitalisation, the NICU staff must adopt flexible and responsive strategies to meet these changing needs. Staff should be sensitive to identify parents' support needs from family and friends, share relevant information and manage the involvement of family and friends in a way that aligns with the parents' preferences.

Moreover, clear and consistent guidelines aligned with FCC principles are necessary to ensure that parents receive the emotional, practical, and social support they need. Practices related to visiting hours and family involvement should be adaptable to reflect parents' evolving needs. Continuous reflection and adaptation of NICU practices are essential to provide comprehensive and personalised care that supports both parents and their support networks throughout the hospitalisation and after discharge.

## Author Contributions

E.K., A.A., H.H.‐T., R.B.J., and R.F. made substantial contributions to conception and design, acquisition of data or analysis and interpretation of data. E.K., A.A., H.H.‐T., R.B.J., and R.F. involved in drafting the manuscript or revising it critically for important intellectual content. E.K., A.A., H.H.‐T., R.B.J., and R.F. given final approval of the version to be published, each author should have participated sufficiently in the work to take public responsibility for appropriate portions of the content. E.K., A.A., H.H.‐T., R.B.J., and R.F. agreed to be accountable for all aspects of the work in ensuring that questions related to the accuracy or integrity of any part of the work are appropriately investigated and resolved.

## Ethics Statement

This study was conducted in accordance with the Declaration of Helsinki, adhering to good scientific practices and principles of research integrity. Participants were provided with information about the study, and participation in interviews was entirely voluntary. Informed written consent was signed by all participants before participation. Ethics approval was obtained from the respective ethical committees in each country involved.

## Conflicts of Interest

The authors declare no conflicts of interest.

## Supporting information


Data S1.

## Data Availability

The data that support the findings of this study are available on request from the corresponding author. The data are not publicly available due to privacy or ethical restrictions.

## References

[jan16707-bib-0001] Adama, E. A. , E. Adua , S. Bayes , and E. Mörelius . 2022. “Support Needs of Parents in Neonatal Intensive Care Unit: An Integrative Review.” Journal of Clinical Nursing 31, no. 5–6: 532–547. 10.1111/jocn.15972. 34312923

[jan16707-bib-0002] Adama, E. A. , S. Bayes , and D. Sundin . 2018. “Parents' Experiences of Caring for Preterm Infants After Discharge With Grandmothers as Their Main Support.” Journal of Clinical Nursing 27, no. 17–18: 3377–3386. 10.1111/jocn.13868. 28474752

[jan16707-bib-0003] Amankwaa, L. C. , R. H. Pickler , and J. Boonmee . 2007. “Maternal Responsiveness in Mothers of Preterm Infants.” Newborn and Infant Nursing Reviews 7, no. 1: 25–30.

[jan16707-bib-0004] Axelin, A. , N. Feeley , M. Campbell‐Yeo , et al. 2022. “Symptoms of Depression in Parents After Discharge From NICU Associated With Family‐Centred Care.” Journal of Advanced Nursing 78, no. 6: 1676–1687. 10.1111/jan.15128. 34897769 PMC9299776

[jan16707-bib-0005] Bowen, M. 1985. Family Therapy in Clinical Practice. Lanham, MD: Jason Aronson.

[jan16707-bib-0006] Bradshaw, C. , S. Atkinson , and O. Doody . 2017. “Employing a Qualitative Description Approach in Health Care Research.” Global Qualitative Nursing Research 4: 2333393617742282. 10.1177/2333393617742282. 29204457 PMC5703087

[jan16707-bib-0007] Braun, V. , and V. Clarke . 2021. “One Size Fits All? What Counts as Quality Practice in (Reflexive) Thematic Analysis?” Qualitative Research in Psychology 18, no. 3: 328–352. 10.1080/14780887.2020.1769238.

[jan16707-bib-0008] Braun, V. , and V. Clarke . 2024. “Doing Reflexive Thematic Analysis.” https://www.thematicanalysis.net/doing‐reflexive‐ta/. 10.1093/heapro/daae049 PMC11132294 38805676

[jan16707-bib-0009] Brødsgaard, A. , T. Helth , B. L. Andersen , and M. Petersen . 2017. “Rallying the Troops: How Sharing Knowledge With Grandparents Supports the Family of the Preterm Infant in Neonatal Intensive Care Unit.” Advances in Neonatal Care: Official Journal of the National Association of Neonatal Nurses 17, no. 3: E1–E10. 10.1097/ANC.0000000000000360. 27902505

[jan16707-bib-0010] de Paula Eduardo, J. A. F. , M. G. de Rezende , P. R. Menezes , and C. M. Del‐Ben . 2019. “Preterm Birth as a Risk Factor for Postpartum Depression: A Systematic Review and Meta‐Analysis.” Journal of Affective Disorders 259: 392–403. 10.1016/j.jad.2019.08.069. 31470184

[jan16707-bib-0011] Ding, X. , L. Zhu , R. Zhang , L. Wang , T.‐T. Wang , and J. M. Latour . 2019. “Effects of Family‐Centred Care Interventions on Preterm Infants and Parents in Neonatal Intensive Care Units: A Systematic Review and Meta‐Analysis of Randomised Controlled Trials.” Australian Critical Care 32, no. 1: 63–75. 10.1016/j.aucc.2018.10.007. 30554939

[jan16707-bib-0012] Finnish National Board on Research Integrity TENK . 2023. “The Finnish Code of Conduct for Research Integrity and Procedures for Handling Alleged Violations of Research Integrity in Finland. Guideline of the Finnish National Board on Research Integrity TENK 2023.” https://tenk.fi/sites/default/files/2023‐05/RI_Guidelines_2023.pdf.

[jan16707-bib-0013] Flacking, R. , C. Breili , and M. Eriksson . 2019. “Facilities for Presence and Provision of Support to Parents and Significant Others in Neonatal Units.” Acta Paediatrica 108, no. 12: 2186–2191. 10.1111/apa.14948. 31350769 PMC6899771

[jan16707-bib-0014] Flacking, R. , H. Haslund‐Thomsen , R. Jónsdóttir , S. Poropudas , and A. Axelin . 2022. “Parents' Friends and Families in Neonatal Intensive Care Units: A Cross‐National Qualitative Study on Staff Perceptions and Experiences.” Journal of Clinical Nursing 31, no. 21–22: 3120–3129. 10.1111/jocn.16139. 34816522

[jan16707-bib-0015] Frisman, G. H. , C. Eriksson , S. Pernehed , and E. Mörelius . 2012. “The Experience of Becoming a Grandmother to a Premature Infant—A Balancing Act, Influenced by Ambivalent Feelings.” Journal of Clinical Nursing 21, no. 21–22: 3297–3305. 10.1111/j.1365-2702.2012.04204.x. 22861154

[jan16707-bib-0016] Hagen, I. H. , V. C. Iversen , E. Nesset , R. Orner , and M. F. Svindseth . 2019. “Parental Satisfaction With Neonatal Intensive Care Units: A Quantitative Cross‐Sectional Study.” BMC Health Services Research 19, no. 1: 37. 10.1186/s12913-018-3854-7. 30646901 PMC6332571

[jan16707-bib-0017] Hall, E. O. C. 2004. “A Double Concern: Grandmothers' Experiences When a Small Grandchild Is Critically Ill.” Journal of Pediatric Nursing 19, no. 1: 61–69. 10.1016/s0882-5963(03)00143-x. 14963873

[jan16707-bib-0018] House, J. S. 1987. “Social Support and Social Structure.” Sociological Forum 2, no. 1: 135–146.

[jan16707-bib-0019] Institute for Patient‐ and Family‐Centered Care . 2024. “Patient‐ and Family‐Centered Care.” https://www.ipfcc.org/about/pfcc.html.

[jan16707-bib-0020] Jones, L. , K. Peters , J. Rowe , and N. Sheeran . 2016. “The Influence of Neonatal Nursery Design on Mothers' Interactions in the Nursery.” Journal of Pediatric Nursing: Nursing Care of Children and Families 31, no. 5: e301–e312. 10.1016/j.pedn.2016.05.005. 27311300

[jan16707-bib-0021] Kainiemi, E. , R. Flacking , L. Lehtonen , M. Pasanen , and A. Axelin . 2022. “Psychometric Properties of an Instrument to Measure the Quality of Family‐Centered Care in NICUs.” Journal of Obstetric, Gynecologic, and Neonatal Nursing 51, no. 4: 461–472. 10.1016/j.jogn.2022.04.004. 35598704

[jan16707-bib-0022] Kainiemi, E. , P. Hongisto , L. Lehtonen , B. Pape , and A. Axelin . 2021. “Effects of Single Family Room Architecture on Parent–Infant Closeness and Family Centered Care in Neonatal Environments—A Single‐Center Pre–Post Study.” Journal of Perinatology 41, no. 9: 2244–2251. 10.1038/s41372-021-01137-z. 34230604 PMC8440171

[jan16707-bib-0023] Kaiser, K. 2009. “Protecting Respondent Confidentiality in Qualitative Research.” Qualitative Health Research 19, no. 11: 1632–1641. 10.1177/1049732309350879. 19843971 PMC2805454

[jan16707-bib-0024] Kallio, H. , A.‐M. Pietilä , M. Johnson , and M. Kangasniemi . 2016. “Systematic Methodological Review: Developing a Framework for a Qualitative Semi‐Structured Interview Guide.” Journal of Advanced Nursing 72, no. 12: 2954–2965. 10.1111/jan.13031. 27221824

[jan16707-bib-0025] Kavanaugh, K. , L. M. Nantais‐Smith , T. Savage , S. M. Schim , and G. Natarajan . 2014. “Extended Family Support for Parents Faced With Life‐Support Decisions for Extremely Premature Infants.” Neonatal Network 33, no. 5: 255–262. 10.1891/0730-0832.33.5.255. 25161133

[jan16707-bib-0026] Kuo, D. Z. , A. J. Houtrow , P. Arango , K. A. Kuhlthau , J. M. Simmons , and J. M. Neff . 2012. “Family‐Centered Care: Current Applications and Future Directions in Pediatric Health Care.” Maternal and Child Health Journal 16, no. 2: 297–305. 10.1007/s10995-011-0751-7. 21318293 PMC3262132

[jan16707-bib-0027] Leep‐Lazar, K. , and A. W. Stimpfel . 2024. “A Dimensional Analysis of Nursing Unit Culture.” Journal of Advanced Nursing 80, no. 7: 2746–2757. 10.1111/jan.15985. 37994224 PMC11109012

[jan16707-bib-0028] Lindberg, B. , and K. Ohrling . 2008. “Experiences of Having a Prematurely Born Infant From the Perspective of Mothers in Northern Sweden.” International Journal of Circumpolar Health 67, no. 5: 461–471. 10.3402/ijch.v67i5.18353. 19186767

[jan16707-bib-0029] Lingard, L. 2019. “Beyond the Default Colon: Effective Use of Quotes in Qualitative Research. Perspectives on.” Medical Education 8, no. 6: 360–364. 10.1007/s40037-019-00550-7. PMC6904374 31758490

[jan16707-bib-0030] McKeown, L. , K. Burke , V. E. Cobham , H. Kimball , K. Foxcroft , and L. Callaway . 2023. “The Prevalence of PTSD of Mothers and Fathers of High‐Risk Infants Admitted to NICU: A Systematic Review.” Clinical Child and Family Psychology Review 26, no. 1: 33–49. 10.1007/s10567-022-00421-4. 36564614

[jan16707-bib-0031] Ravindran, V. P. , and G. R. Rempel . 2011. “Grandparents and Siblings of Children With Congenital Heart Disease.” Journal of Advanced Nursing 67, no. 1: 169–175. 10.1111/j.1365-2648.2010.05482.x. 21039778

[jan16707-bib-0032] Rossman, B. , M. M. Greene , and P. P. Meier . 2015. “The Role of Peer Support in the Development of Maternal Identity for “NICU Moms”.” Journal of Obstetric, Gynecologic & Neonatal Nursing 44, no. 1: 3–16. 10.1111/1552-6909.12527. PMC4315745 25580732

[jan16707-bib-0033] Shimizu, A. , and A. Mori . 2018. “Maternal Perceptions of Family‐Centred Support and Their Associations With the Mother‐Nurse Relationship in the Neonatal Intensive Care Unit.” Journal of Clinical Nursing 27, no. 7–8: e1589–e1599. 10.1111/jocn.14243. 29266474

[jan16707-bib-0034] Smith, V. C. , G. K. Steelfisher , C. Salhi , and L. Y. Shen . 2012. “Coping With the Neonatal Intensive Care Unit Experience: Parents' Strategies and Views of Staff Support.” Journal of Perinatal & Neonatal Nursing 26, no. 4: 343–352. 10.1097/JPN.0b013e318270ffe5. 23111723

[jan16707-bib-0035] Tong, A. , P. Sainsbury , and J. Craig . 2007. “Consolidated Criteria for Reporting Qualitative Research (COREQ): A 32‐Item Checklist for Interviews and Focus Groups.” International Journal for Quality in Health Care: Journal of the International Society for Quality in Health Care 19, no. 6: 349–357. 10.1093/intqhc/mzm042. 17872937

[jan16707-bib-0036] Wigert, H. , M. B. Dellenmark , and K. Bry . 2013. “Strengths and Weaknesses of Parent–Staff Communication in the NICU: A Survey Assessment.” BMC Pediatrics 13, no. 1: 71. 10.1186/1471-2431-13-71. 23651578 PMC3651269

