# Supplementary material for: Parents' Need for Support From Family and Friends During Their Preterm Infants' Hospitalisation: A Cross‐National Qualitative Study
Source: J Adv Nurs. 2024 Dec 30;81(8):4989–99. doi: 10.1111/jan.16707 (PMC12271666; doi:10.1111/jan.16707)
Supplement: Supplementary file 1 — Data S1. [file JAN-81-4989-s001.docx]

**Supplementary material**

**Interview guide for interviews with parents**

Initial description

- Short presentation of the one interviewing
- Aim of the study
- All that is said is of interest – there are no right/wrong things to be said
- Use of taperecorder – ok to use?
- Can interrupt the interview at any time
- All that is said is confidential

Questions

- Tell me a bit about why you are here?
- What kind of support do you feel that you have received so far?
  - Grandparents
  - Relatives
  - Friends

Why do you think that you have received that (or not)? (to explore the question above)

- What types of support have you lacked and needed?
  - Grandparents
  - Relatives
  - Friends
- Do they know that you needed that support? (explore further on why)
- Could you try to describe what kind of support you would like from those that you would like support from: what practical (inside and outside of the unit), social, and emotional support would you appreciate?
  - Grandparents
  - Relatives
  - Friends

What can you see as hindrances for getting the support you want/need?

What needs to be “in place” for getting the support you want/need?

- What types of support (practical support inside and outside of the unit, social and emotional support) would you not like to have?
  - Grandparents
  - Relatives
  - Friends

What risks can you see for getting support that you don’t want? And how can those risks be reduced?

- On a more general level – what kind of role would you like them to have for you, your partner and your baby?
  - Grandparents
  - Relatives
  - Friends
